# Supplementary material for: Annual aboveground carbon uptake enhancements from assisted gene flow in boreal black spruce forests are not long-lasting
Source: Nat Commun. 2021 Feb 19;12:1169. doi: 10.1038/s41467-021-21222-3 (PMC7895975; doi:10.1038/s41467-021-21222-3)
Supplement: Supplementary file 1 — Supplementary Information [file 41467_2021_21222_MOESM1_ESM.pdf]

Supplementary Information

Annual aboveground carbon uptake enhancements from assisted gene flow in boreal black spruce forests are not long-lasting

Martin P. Girardin[1, 2], Nathalie Isabel[1,3], Xiao Jing Guo[1], Manuel Lamothe[1], Isabelle Duchesne[4], Patrick Lenz[3,4]

1. Natural Resources Canada, Canadian Forest Service, Laurentian Forestry Centre, 1055 du P.E.P.S, P.O. Box 10380, Stn. Sainte-Foy, Québec, QC, G1V 4C7, Canada
2. Centre d'étude de la forêt, Université du Québec à Montréal, C.P. 8888, succ. Centre-ville, Montréal, QC, H3C 3P8, Canada
3. Canada Research Chair in Forest Genomics, Faculté de Foresterie, de Géographie et de Géomatique, Université Laval, Québec, QC G1V 0A6, Canada
4. Natural Resources Canada, Canadian Wood Fibre Centre, 1055 du P.E.P.S, P.O. Box 10380, Stn. Sainte-Foy, Québec, QC, G1V 4C7, Canada

Supplementary Figures

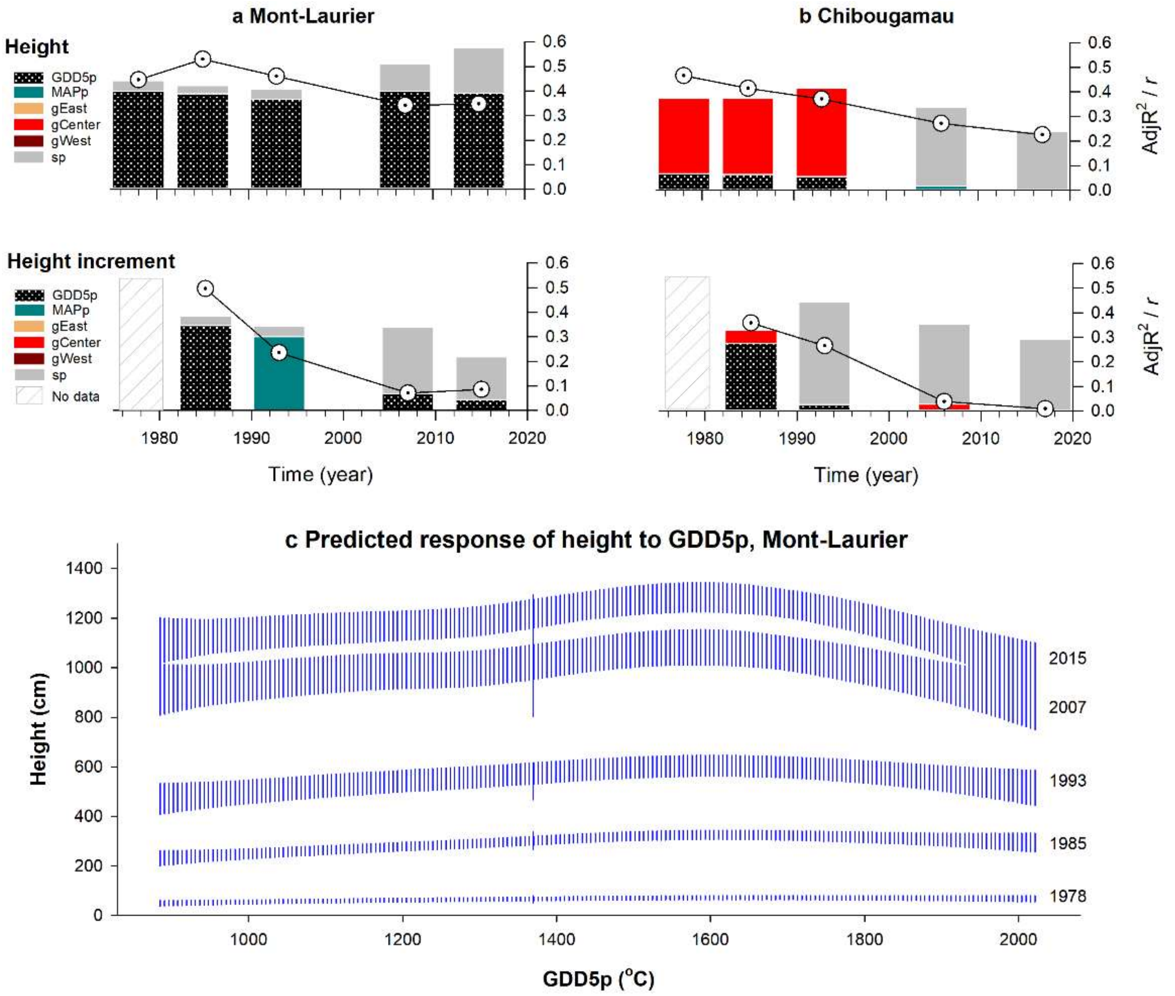

Fig. 1 Test for population differentiation and clinal variations in field-based measurements of tree height and tree height increment. Under competitive effects, differences in height growth should exist beyond 15 years after planting, while increment differences should stabilize. To test this hypothesis, we analysed the field-based height and height increment data at the **a** Mont-Laurier and **b** Chibougamau common gardens. A high adjusted R-squared (adjR^2) value denotes a high goodness-of-fit between tree height and the explanatory variables GDD5p, mean annual precipitation (MAPp), admixture proportions along *Western*, *Central*, and *Eastern* genetic clusters (gWest, gCenter, gEast), and spatial factors (sp) represented by the distance-based Moran's eigenvector maps. The dotted-circled lines illustrate the Pearson correlation *r* between height variables and GDD5p. **c** Generalized Additive Model (GAM) predictions (two-sided 95% confidence intervals) of the relationship between GDD5p and height at the Mont-Laurier site. The number of sampled independent population was *n*=42 for Mont-Laurier and *n*=45 for Chibougamau, each having three replicates. The relationship between clinal variations and field-based measurements of tree height in Mont-Laurier clearly persists for a period beyond 15 years, while it diminishes for height increments. Interestingly, the high adjR^2 between GDD5p and height

is a consequence of the GAM capturing a break in the linear effect, with the provenances from high GDD5p having the greatest height in early censuses (1978, 1985) switching to having amongst the lowest height in the most recent census (2015). The pattern is less clear at Chibougamau, mainly because the spatial factors are seemingly better predictors of height than GDD5p in the last two censuses. But we do see a reduction of the predictive power of the center lineage. We also see a pattern similar to that of Mont-Laurier when analysing the linear correlation between GDD5p and the height variables: the correlation between GDD5p and field-based measurements of tree height persists for a period beyond 15 years, while it diminishes when looking at height increment (dotted-circled lines).

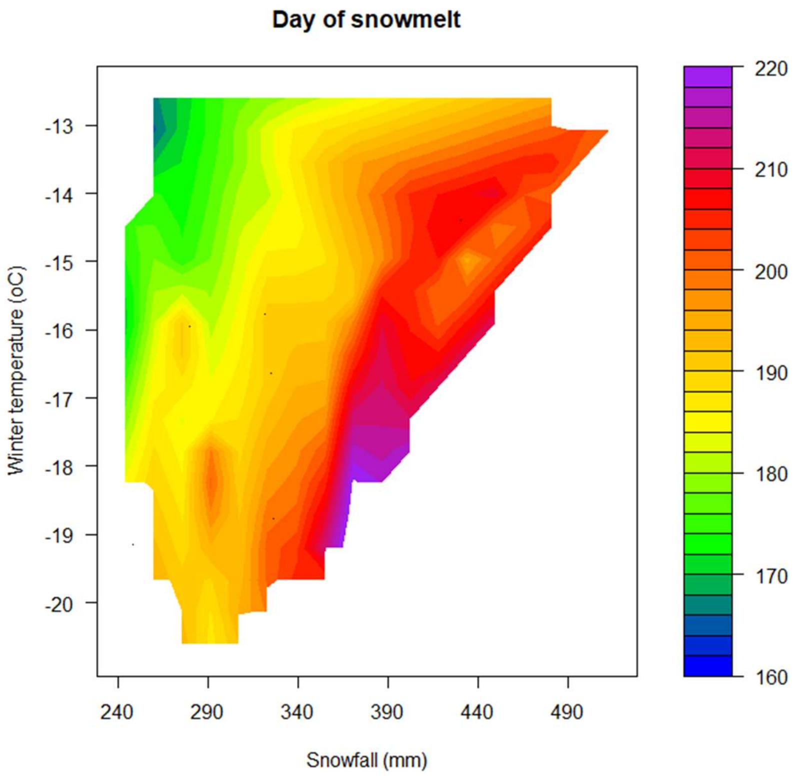

Fig. 2 Relationship between the amount of snowfall (in mm), winter temperatures (°C), and the timing of snowmelt (day of year). The relationship was estimated for Chibougamau's common garden using the BioSIM software, period 1984-2016. A linear interpolation to the irregularly gridded snowmelt data was applied in the snowfall and winter temperature dimensions using the Akima R package[1,2].

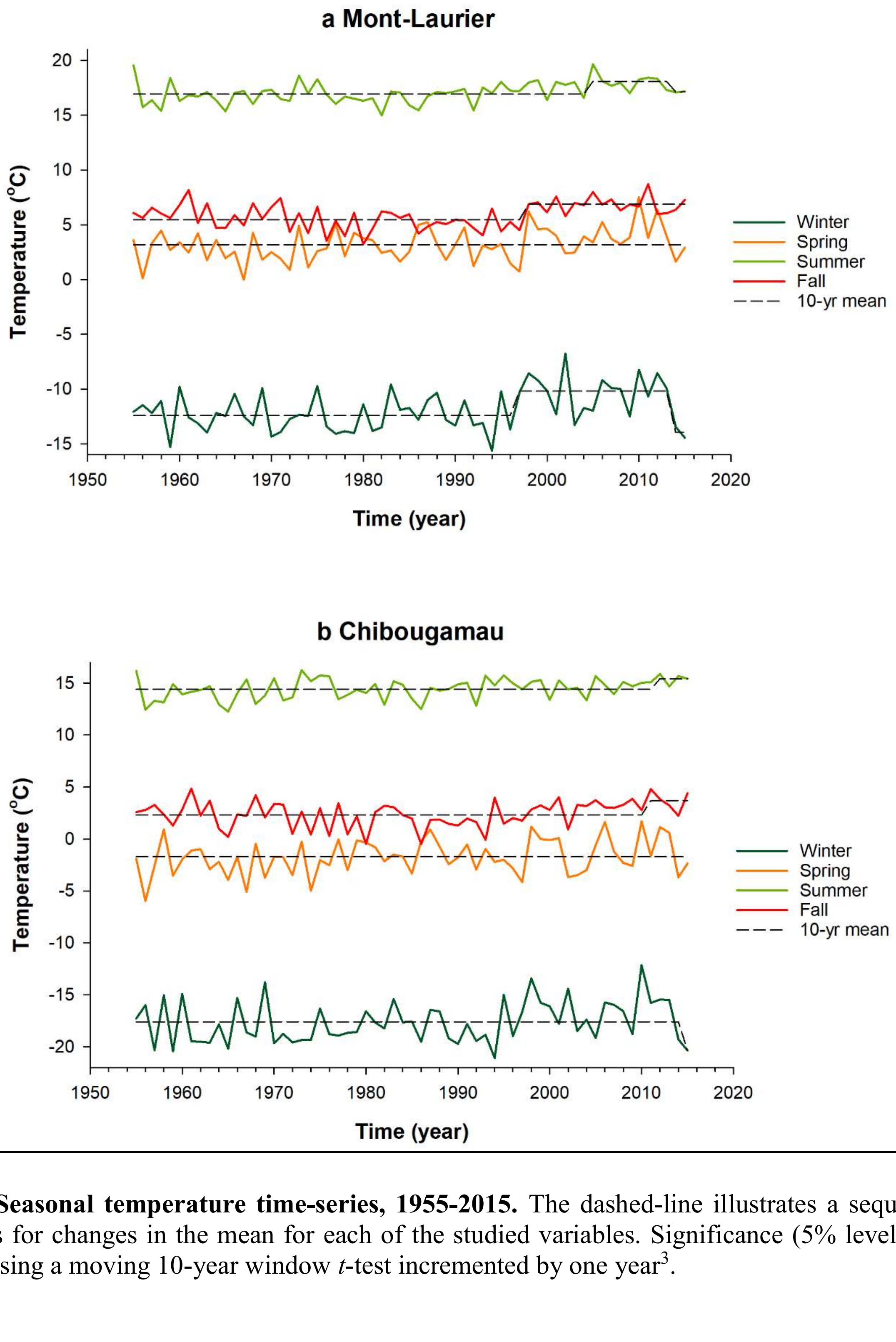

Fig. 3 Seasonal temperature time-series, 1955-2015. The dashed-line illustrates a sequential analysis for changes in the mean for each of the studied variables. Significance (5% level) was tested using a moving 10-year window *t*-test incremented by one year[3].

Supplementary Tables

Table 1 Genetic parameters and pairwise F_{ST} estimated for each lineage (k=3).

					Pairwise F_{ST}*	
Lineages	n	Ho	Hs	Fis	*CENTRAL*	*EASTERN*
Central	696	0.349	0.371	0.059	.	.
Eastern	653	0.342	0.363	0.058	0.014	.
Western	279	0.297	0.313	0.054	0.067	0.087

*all Pairwise FST are significant at $p < 0.001$

Table 2 Results of the Mantel test. We tested if similarities among population-by-block time-series of NPP_{gpbt} in a given garden were associated with the geographic distances between provenances. Starting with the first block of the Mont-Laurier site, Pearson correlations r were computed between pairwise combinations of the block NPP_{gpbt} time-series; results were assembled in the form of a distance matrix. This step was repeated on the second and third blocks. Mantel tests were then conducted separately for each block.

**Mont-Laurier**		
**Bloc**	***r***	***P***
3	0.26	0.021
4	-0.02	0.578
6	0.11	0.065
**Chibougamau**		
**Bloc**	***r***	***P***
3	0.33	0.018
4	-0.04	0.707
5	0.31	0.009

Table 3 Generalized Additive Mixed Models (GAMM) R-squared values for the fit between annual net primary productivity (NPP) and explanatory climate variables, by provenance and experimental site. Refer to Fig. 3 and Equation (5) in main text for additional model statistics.

Provenance	Mont-Laurier R-squared	Chibougamau R-squared
321	0.962	0.988
325	0.959	0.973
326	0.953	0.979
329	0.978	0.980
332	0.969	0.981
333	0.973	0.980
336	0.967	0.982
338	0.953	0.989
342	0.965	0.974
345	0.966	0.988
352	0.961	0.979
355	0.958	0.981
369	0.974	0.981
1329	0.955	0.986
1528	0.966	0.979
1530	0.973	0.982
1531	0.965	0.979
1534	0.957	0.982
1538	0.966	0.988
3268	0.938	0.972
4277	0.965	0.972
4344	0.962	0.981
4351	0.963	0.980
4353	0.968	0.979
4360	0.971	0.980
6801	0.970	0.976
6802	0.961	0.975
6804	0.967	0.985
6805	0.970	0.980
6901	0.961	0.985
6907	0.965	0.989
6909	0.978	0.985
6914	0.982	0.983
6917	0.976	0.986
6922	0.975	0.982
6927	0.966	0.983
6930	0.953	0.985
6938	0.961	0.986
6965	0.960	0.978
6967		0.982
6968	0.979	
6970		0.980
6973	0.965	0.984
6979	0.976	0.974
6986		0.973
7000		0.976

Table 4 Summary of ordinary least-squares linear trend models on seasonal temperature, precipitation, and soil moisture index variables over the period 1975–2015. Variables for which a significant linear trend was detected, as tested using the Spearman rank correlation *r* with $P < 0.050$, are identified in bold.

	Mont-Laurier				Chibougamau			
**Variables**	**Mean**	**Slope**	***r***	***P***	**Mean**	**Slope**	***r***	***P***
**Temperature**								
Winter	**-11.6**	**0.056**	**0.34**	**0.03**	-17.4	0.038	0.21	0.185
Spring	3.5	0.026	0.2	0.201	-1.4	0.009	0.04	0.812
Summer	**17.2**	**0.04**	**0.55**	**0**	14.6	0.021	0.28	0.074
Fall	**5.9**	**0.063**	**0.62**	**0**	**2.4**	**0.057**	**0.52**	**0.001**
**Precipitation**								
Winter	200.6	0.232	0.02	0.898	163.6	0.56	0.23	0.148
Spring	219.7	-0.427	-0.02	0.884	186.2	-0.186	-0.01	0.947
Summer	295.7	0.744	0.12	0.469	315.6	-0.004	-0.01	0.945
Fall	278.8	0.398	0.1	0.546	**284.6**	**0.252**	**0.11**	**0.499**
**Soil Moisture Index (SMI)**								
Spring	98.3	-0.028	0.02	0.884	98.9	-0.067	-0.17	0.28
Summer	92.3	0.077	0.19	0.234	96.3	-0.039	-0.18	0.266
Fall	95.4	0.042	-0.09	0.562	**99**	**-0.033**	**-0.43**	**0.006**
**Snowfall**	268.4	-0.777	-0.23	0.142	313.4	0.04	-0.02	0.915

Supplementary References

1. Akima, H. A Method of Bivariate Interpolation and Smooth Surface Fitting for Irregularly Distributed Data Points. *ACM Trans. Math. Softw.* **4**, 148–159 (1978).
2. R Development Core Team. R: A Language and Environment for Statistical Computing. (2013).
3. Rodionov, S. N. A sequential algorithm for testing climate regime shifts. *Geophysical Research Letters* **31**, L09204 (2004).
