# Supplementary material for: Annual aboveground carbon uptake enhancements from assisted gene flow in boreal black spruce forests are not long-lasting
Source: Nat Commun. 2021 Feb 19;12:1169. doi: 10.1038/s41467-021-21222-3 (PMC7895975; doi:10.1038/s41467-021-21222-3)
Supplement: Supplementary file 3 — Description of Additional Supplementary Information [file 41467_2021_21222_MOESM3_ESM.pdf]

Description of Additional Supplementary Files

File Name: Supplementary Data 1

Description: Mean annual bioclimatic characteristics of the provenances over the 1961-1990 period. MAT: mean annual temperature; MAP: mean annual precipitation; GDD5: growing degree days >5°C; Radiation: total annual radiation. For each provenance, averaged admixture proportions (in %, K =3) for Western, Central, and Eastern lineages (gWest, gCenter, gEast) are provided.

File Name: Supplementary Data 2

Description: Sample distribution for DNA extraction from frozen needle tissues. A total of 1,628 trees (n) from 67 provenances were genotyped for 239 known SNPs of the P. mariana species. To obtain a better coverage of BS diversity and population structure, and to correctly assign a specific population to its genetic lineage, we added trees from three other common garden sites (namely Petawawa, Acadia, Valcartier), part of the same range-wide provenance study. Provenances that are unique to these gardens are highlighted in grey. For each provenance, admixture proportions (in %) for western, central, and eastern lineages (gWest, gCenter, gEast) are provided.

File Name: Supplementary Data 3

Description: Total carbon (TotalC) by provenance and common garden, as of the years 1984 and 2015. A rank was applied to each provenance and year based on performance (TotalC). The five highest values are highlighted in gray for each garden and year.
