# Supplementary material for: Annual aboveground carbon uptake enhancements from assisted gene flow in boreal black spruce forests are not long-lasting
Source: Nat Commun. 2021 Feb 19;12:1169. doi: 10.1038/s41467-021-21222-3 (PMC7895975; doi:10.1038/s41467-021-21222-3)
Supplement: Supplementary file 7 — Reporting Summary [file 41467_2021_21222_MOESM7_ESM.pdf]

nature research

Corresponding author(s): Martin P. Girardin

Last updated by author(s): Dec 22, 2020

Reporting Summary

Nature Research wishes to improve the reproducibility of the work that we publish. This form provides structure for consistency and transparency in reporting. For further information on Nature Research policies, see our Editorial Policies and the Editorial Policy Checklist.

Statistics

For all statistical analyses, confirm that the following items are present in the figure legend, table legend, main text, or Methods section.

n/a	Confirmed	
☐	☒	The exact sample size (*n*) for each experimental group/condition, given as a discrete number and unit of measurement
☐	☒	A statement on whether measurements were taken from distinct samples or whether the same sample was measured repeatedly
☐	☒	The statistical test(s) used AND whether they are one- or two-sided<br>*Only common tests should be described solely by name; describe more complex techniques in the Methods section.*
☐	☒	A description of all covariates tested
☐	☒	A description of any assumptions or corrections, such as tests of normality and adjustment for multiple comparisons
☐	☒	A full description of the statistical parameters including central tendency (e.g. means) or other basic estimates (e.g. regression coefficient) AND variation (e.g. standard deviation) or associated estimates of uncertainty (e.g. confidence intervals)
☐	☒	For null hypothesis testing, the test statistic (e.g. F, t, r) with confidence intervals, effect sizes, degrees of freedom and P value noted<br>*Give P values as exact values whenever suitable.*
☒	☐	For Bayesian analysis, information on the choice of priors and Markov chain Monte Carlo settings
☐	☒	For hierarchical and complex designs, identification of the appropriate level for tests and full reporting of outcomes
☐	☒	Estimates of effect sizes (e.g. Cohen's *d*, Pearson's *r*), indicating how they were calculated

Our web collection on statistics for biologists contains articles on many of the points above.

Software and code

Policy information about availability of computer code

Data collection	- Acquisition of annual rings of each core was done using the software Coo-Recorder (https://www.cybis.se/).<br>- Acquisition of ring density profiles was done using the software provided with the Quintek X-Ray measuring system (http://www.qms-density.com/).<br>- Trees were genotyped for known SNPs using the Sequenom iPLEX Gold technology.
Data analysis	- Verification of cross-dating of annual rings of each core was done using the softwares Coo-Recorder 8.11 (https://www.cybis.se/) and COFECHA 6.06 (https://www.ldeo.columbia.edu/tree-ring-laboratory/resources/software).<br>- Weather data were 'statistically' interpolated from observed data using the BioSIM 10.3.2 software (https://cfs.nrcan.gc.ca/projects/133).<br>- Function SMI in BioSIM 10.3.2 was used for computation of the soil moisture index. Parameter settings are provided in text.<br>- Inference of genetic structure was made using Structure v2.3.4 (https://web.stanford.edu/group/pritchardlab/structure_software/release_versions/v2.3.4/html/structure.html).<br>- Genetic diversity calculation by lineage, pairwise FST between the lineages, and standard hierarchical analysis of the molecular variance, have all been performed using Genodive v3.04 (http://www.patrickmeirmans.com/software/GenoDive.html).<br>- Statistical analyses were performed in the R environment v3.5.3 (R Development Core Team) using existing functions (mantel.rtest) and packages (mgcv 1.8-4, spmoran 0.2.0-2).<br>- Maps were created in ArcGIS version 10.5.1 (www.arcgis.com).<br>- Figures were drafted in SigmaPlot version 14 (https://systatsoftware.com/products/sigmaplot/).

For manuscripts utilizing custom algorithms or software that are central to the research but not yet described in published literature, software must be made available to editors and reviewers. We strongly encourage code deposition in a community repository (e.g. GitHub). See the Nature Research guidelines for submitting code & software for further information.

April 2020

Data

Policy information about availability of data

All manuscripts must include a data availability statement. This statement should provide the following information, where applicable:

- Accession codes, unique identifiers, or web links for publicly available datasets
- A list of figures that have associated raw data
- A description of any restrictions on data availability

Data that support the results of this study are available on figshare (https://doi.org/10.6084/m9.figshare.13420802.v1). Weather data are freely accessible through Environment Canada's portal (https://climate.weather.gc.ca/) and the BioSIM server (https://cfs.nrcan.gc.ca/projects/133). Source data for Figs. 1, 2, 3, 5 and 6 and Supplementary Fig. 1 are provided with this paper.

All relevant software and R-functions that were used in this paper are referred to in the methods section (see package vignettes for details). Custom codes are available on figshare (https://doi.org/10.6084/m9.figshare.13420802.v1).

Field-specific reporting

Please select the one below that is the best fit for your research. If you are not sure, read the appropriate sections before making your selection.

☐ Life sciences ☐ Behavioural & social sciences ☒ Ecological, evolutionary & environmental sciences

For a reference copy of the document with all sections, see nature.com/documents/nr-reporting-summary-flat.pdf

Ecological, evolutionary & environmental sciences study design

All studies must disclose on these points even when the disclosure is negative.

Study description

We provide an assessment of the potential of assisted gene flow in the context of climate mitigation with a study of 46 populations representative of the widespread boreal conifer Picea mariana. Our framework takes into account the demographic history of the species, the responses of individual genotypes to climate fluctuations along a tree's lifetime, and the effect of the resulting tree survival/mortality rates. Using a dendroecological approach, we conducted a retrospective analysis of phenotypic variability in annual aboveground net primary productivity (NPP) and looked for signals of local adaptation and/or the presence of phenotypic clines across species and tree lifespan by also taking into account the phylogeographic structure. We also assessed genotype-by-environment interactions by evaluating climate-NPP relationships across populations and common gardens. Our assessment suggests that local adaptation to climate of origin and belonging to genetic lineages had an influence on NPP for a period of approximately 15 years after planting, after which there was little to no effect.

Research sample

We use the term “provenance” when referring to the geographic and climatic origin of a population, and the term “population” when referring to the trees grown from seed sampled at the provenance level (one population per provenance). We examined 51,029 tree rings from 1,560 trees growing since 1974 in two common gardens located at Mont-Laurier and Chibougamau. The common garden experiments were established in contrasting Canadian boreal forest regions, with the southern site (Mont-Laurier) being 4.9 ºC warmer than the northern site (Chibougamau). The design of the gardens consisted of a completely randomized experiment with six blocks, in which 16-tree (4 × 4) square plots were established for each of the seed provenances, with trees spaced at 2.45 m × 3.05 m at the Mont-Laurier site and 2.40 m × 2.40 m at the Chibougamau site. For the current study, three blocks were randomly chosen for sampling of 42 and 45 provenances for the Mont-Laurier and Chibougamau sites, respectively. Forty-one provenances were common to both sites. Having this amount of provenances and blocks per provenances ensured having sufficient amounts of replicates in later statistical analyses.

Sampling strategy

The selection of provenances was made so as to maximize the spatial representativeness of the species distribution while minimizing local redundancy in the seed sources. Three plot replicates are provided for each provenance. In each plot, 6 to 7 living trees were selected for sampling in order to estimate the NPP composite population phenotype. This amount of sampled trees per plot is the typical sample size per plot collected for forest inventories. The four trees in the plot center were prioritized, with additional trees sampled being the largest trees of the plot. A 5-mm-diameter increment core was taken (from bark to pith) from each tree at 1.3 m above ground using a Pressler increment borer. Foliage from the upper-third of the living crown was also collected for DNA analyses. Together, our sampling gives a robust measure of population phenotypic variability,

Data collection

Field data were collected by the field technicians (Acknowledgement section) of the Laurentian Forestry Center, Canadian Forest Service, under the supervision of PL, ID, and NI. Data acquired by laboratory work were produced by lab technicians (Acknowledgement section) of the Laurentian Forestry Center, Canadian Forest Service, under the supervision of M.P.G., N.I., I.D., and P.L. Information regarding tree mortality or unusual tree conditions were obtained during sampling campaigns, along with tree diameter at breast height and tree height for each of the trees present in each plot. These inventory data were compiled with those collected during censuses carried out in the past by our partners (Acknowledgement section). Pressler increment borers were used for sampling of tree cores. Annual rings of each core were aquired using the software Coo-Recorder v8.11. Trees were genotyped for by the Génome Québec Innovation Centre genotyping platform (McGill University, Montréal, Canada) using the Sequenom iPLEX Gold technology.

Timing and spatial scale

The start and end dates of the data coverage are limited to the establishment of the experiments in the 1970s and the time of collection. Sampling was conducted in the autumns of 2015 for the Mont-Laurier and 2016 for the Chibougamau sites. The data from the two common gardens hence consists of annually resolved radial growth and ring density time-series covering the period 1976 to 2015/2016 for each sampled tree and block for 46 black spruce populations representative of the species range .

Data exclusions	No data were excluded from our analyses.
Reproducibility	All measurements were taken in three block replicates for each population from two sites that perform independently.
Randomization	The design of the gardens consisted of a completely randomized experiment with six blocks, in which 16-tree (4 × 4) square plots were established for each of the seed provenances. For the current study, three blocks were randomly chosen for sampling of provenances. Blocks are included as a random effect in statistical analyses.
Blinding	Blinding was not relevant to our study because we did not use experimental methods. Because we make use of observational time-series datasets, analyses were performed in a way to remove time-series biases.

Did the study involve field work? ☒ Yes ☐ No

Field work, collection and transport

Field conditions	Field work was carried in the fall. There were not particular condition during filed work that needed to be taken into account for the current study.
Location	The study took place in two common garden sites, one established near the city of Mont-Laurier, Québec (Canada, 46.36°N, 75.48° W, elev. 244m) and the other near the city of Chibougamau (Canada, 50.18°N, 74.18°W, elev. 411m).
Access & import/export	Common gardens are accessible; no special permit is required.
Disturbance	Information regarding tree mortality or unusual tree conditions were noted. Tree coring was done in the current study, as it is considered as not damaging to trees.

Reporting for specific materials, systems and methods

We require information from authors about some types of materials, experimental systems and methods used in many studies. Here, indicate whether each material, system or method listed is relevant to your study. If you are not sure if a list item applies to your research, read the appropriate section before selecting a response.

Materials & experimental systems

n/a	Involved in the study	
☒	☐	Antibodies
☒	☐	Eukaryotic cell lines
☒	☐	Palaeontology and archaeology
☒	☐	Animals and other organisms
☒	☐	Human research participants
☒	☐	Clinical data
☒	☐	Dual use research of concern

Methods

n/a	Involved in the study	
☒	☐	ChIP-seq
☒	☐	Flow cytometry
☒	☐	MRI-based neuroimaging
